# The efficacy and safety of remifentanil patient-controlled versus epidural analgesia in labor: A meta-analysis and systematic review

**DOI:** 10.1371/journal.pone.0275716

**Published:** 2022-12-19

**Authors:** Xiuzhen Lei, Yang Yu, Mei Li, Peng Fang, Shuyuan Gan, Yongxing Yao, Yanfeng Zhou, Xianhui Kang

**Affiliations:** Department of Anesthesiology, The First Affiliated Hospital, School of Medicine, Zhejiang University, Hangzhou, Zhejiang, China; University of Palermo: Universita degli Studi di Palermo, ITALY

## Abstract

**Background:**

Remifentanil patient-controlled analgesia (rPCA) and epidural analgesia (EA) has been used for pain relief in labor. We aimed to evaluate the efficacy and safety of rPCA versus EA in labor, to provide evidence support for clinical analgesia and pain care.

**Methods:**

We searched PubMed, EMBASE, ScienceDirect, Cochrane Library, China National Knowledge Infrastructure (CNKI), Wanfang and Weipu databases for RCTs comparing rPCA and EA in labor until February 15, 2022. Two researchers independently screened literature and extracted data. RevMan 5.3 software was used for data analysis.

**Results:**

A total of 10 RCTs involving 3086 parturients were enrolled, 1549 parturients received rPCA and 1537 received EA. Meta-analysis indicated that the incidence of intrapartum maternal fever within 1 hour of labor analgesia (OR = 0.43, 95%CI: 0.30~0.62), after 1 hour of labor analgesia (OR = 0.42, 95%CI: 0.20~0.90) in the rPCA was significantly less than that of EA (all P<0.05). The incidence of respiratory depression (OR = 3.56, 95%CI: 2.45~5.16, P<0.001) in the rPCA was significantly higher than that of EA. There were no significant differences in the incidence of Apgar scores<7 at 5 minutes (OR = 1.18, 95%CI: 0.71~1.96, P = 0.53), the patients’ satisfaction of pain relief during labor analgesia (SMD = 0.03, 95%CI: -0.40~0.46, P = 0.90) between rPCA and EA (all P>0.05).

**Conclusion:**

rPCA can be an optional alternative to EA with similar pain relief and less risk of intrapartum maternal fever. However, rPCA was associated with increased risk of respiratory depression. Future studies with rigorous design and larger sample size are needed to provide more reliable evidences for clinical rPCA and EA use.

## Background

With the rapid development of medical science, and the increasing demands of modern people on the pain relief and quality of life, labor analgesia has been paid more and more attention by mothers and medical workers [1]. At present, the main methods of labor analgesia include spinal analgesia, application of sedative drugs such as pethidine and diazepam, and some non-drug labor analgesia, such as water birth, etc., of which spinal analgesia accounts for more than half of the analgesia [2]. Spinal analgesia is now very mature and is the gold standard for labor analgesia in the world. But spinal anesthesia is often accompanied by some deficiencies since it is an invasive operation with certain risks including prolonging the second stage of labor, and dizziness, nausea and vomiting may occur during the process [3, 4]. Therefore, it is of great significance to seek effective and safe methods of labor analgesia.

Epidural anesthesia (EA), as one of the most common methods of labor analgesia, has the advantages of strong analgesic effect, fixed analgesic plane, long duration, and easy control of drug dosage [5, 6]. It can effectively relieve labor pain, and the effect is stronger than that of general opioid anesthesia [7, 8]. However, studies [9, 10] have found that EA can prolong the second stage of labor. As a pure opioid u-type receptor agonist, remifentanil has the characteristics of fast onset, short duration of action, and fast metabolic rate [11, 12]. At the same time, remifentanil does not increase the time of the second stage of labor. Several randomized controlled trials (RCTs) have compared the applications of remifentanil patient-controlled analgesia (rPCA) and EA in labor, yet the results remain inconsistent or even conflicting. Therefore, we aimed to perform a meta-analysis and systematic review to evaluate the efficacy and safety of rPCA and EA in labor, to provide reliable evidence to the clinical analgesia management in labor.

## Methods

This present meta-analysis and systematic review was performed and reported in accordance to the guidelines of the Preferred Reporting Items for Systematic Reviews and Meta-analysis (PRISMA) statement [13]. Ethics approval and consent to participate is not necessary since our study is a meta-analysis and systematic review.

### Inclusion and exclusion criteria

The inclusion criteria of RCT for this meta-analysis were as follows (1) study design: RCT comparing the effects of rPCA and EA in labor; (2) Research population: healthy nulliparous or parous women with single or multiple gestations; (3) The RCT reported the corresponding outcome data such as the incidence of intrapartum maternal fever(body temperature≥38.5°), patients’ satisfaction of pain relief(0 to 50 scale), fetal respiratory depression(fetal heart rate ≥ 180 beats per minute, or ≤ 110 beats per minute, and less than 10 fetal movements in 12 hours), and the data can be extracted; (4) The RCT was published and reported in the language of English or Chinese.

The literature exclusion criteria for this meta-analysis were as follows: (1) RCTs focused on alternate use of two or more analgesic methods; (2) Duplicate publications; (3) Reviews, editorials, letters or case reports.

### Literature search

Two investigators performed a scientific literature search on PubMed, EMBASE, ScienceDirect, Cochrane Library, China National Knowledge Infrastructure (CNKI), Wanfang and Weipu databases. The following keywords o and corresponding Medical Subject Headings (MeSH) were used for search in every database: ("remifentanil" OR "remifentanil patient-controlled analgesia") AND ("epidural analgesia" OR "labour analgesia" OR "labor analgesia" OR "painless labor" OR "painless labour" OR "painless delivery". At the same time, we manually searched the references of the included RCTs and important reviews. The search time limit was from the establishment of the database to February 15, 2022, and the search languages were limited in English and Chinese.

### Literature screening and data extraction

Two researchers independently screened literature and extracted data. When there was disagreement between the two researchers, the third researcher made the decision. The evaluated indicators in this meta-analysis included: RCT characteristics including country, population, maternal age, gestational age, and details of labor analgesia regimen, related outcome data including intrapartum maternal fever, pain relief, Apgar scores and complications.

### Quality evaluation of included RCT

The Cochrane Collaborations tool [14] of risk of bias was adopted by two investigator independently to assess the study quality and risk of bias of included RCTs. Any disagreements were resolved by further discussion and consensus. Seven specific domains were evaluated with this tooll: sequence generation, allocation concealment, blinding of participants and personnel, blinding of outcome assessment, incomplete outcome data, selective outcome reporting and other issues. Every domain was rated as low risk of bias, high risk of bias or unclear risk of bias according to the judgment criteria.

### Statistical processing

We used the RevMan 5.3 evaluation software provided by the Cochrane Collaboration for analysis. The heterogeneity among the studies was analyzed by the Q test. If I^2^≤50% indicated no heterogeneity among the studies, a fixed effect model was used for data synthesis. If there is heterogeneity between studies(I^2^≤50%), we analyzed the reasons for the heterogeneity, and random effects model was used for data synthesis; Continuous variable effect indicators were expressed by standard mean difference (SMD) and its 95% confidence interval (CI), and dichotomous variable effect indicators were expressed by odd ratio (OR) and its 95% CI. In this meta-analysis, P<0.05 was considered as a statistically significant difference between groups.

## Results

### RCT selection

**Fig 1:**
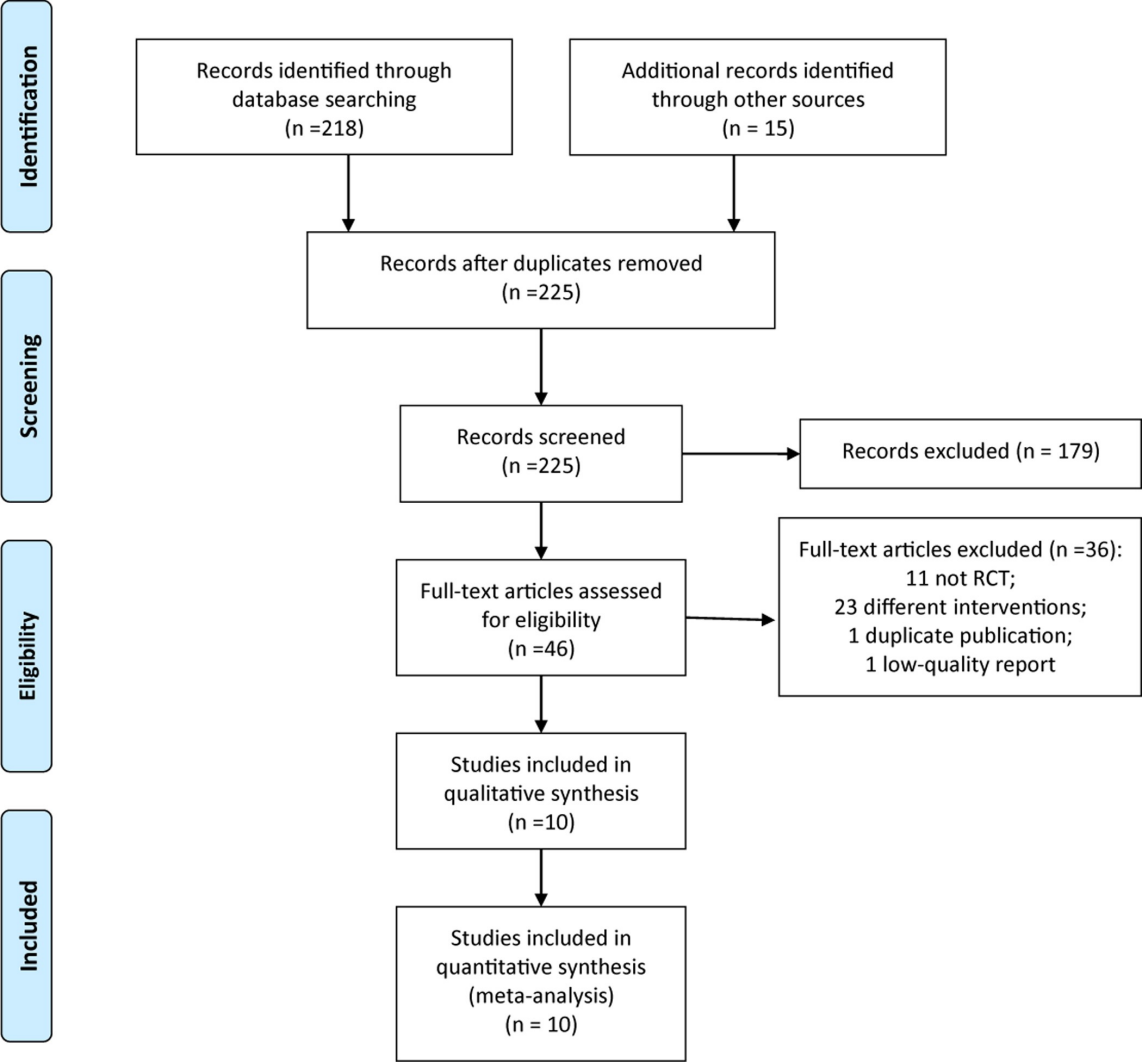
The PRISMA flow diagram of study selection.

The process of RCT selection is presented in Fig 1. We initially identified 233 reports through database searching, we excluded 179 reports based on titles and abstracts. After reviewing 46 full-text reports, we further excluded 36 trials that did not meet the inclusion criteria. Eventually, 10 RCTs [15–24] were included in this present meta-analysis.

### The characteristics of included RCTs

Of the included 10 RCTs [15–24], a total of 3086 parturients were enrolled and evaluated, of whom 1549 parturients received rPCA and 1537 received EA. All included RCTs were published between 2008 and 2019. All the included patients did not use other systemic opioids. The detailed characteristics of 10 included RCTs are presented in Table 1.

**Table 1:** The characteristics of included RCTs.

RCT	Country	Sample size		Maternal age (y)		Number of births (nulliparous vs multiparous)	Gestations (single vs multiple)	Gestational age(weeks)		Intervention		Measure duration
		rPCA	EA	rPCA	rPCA			rPCA	rPCA	rPCA	rPCA	
Douma 2015	Netherlands	49	49	32±4.8	31±5.6	Both	Both	39	40	Bolus:40 μg lockout time: 2 min no background infusion	Loading dose:25 mg (12.5 ml ropivacaine 0.2%) CI: ropivacaine 0.1% plus sufentanil 0.5 μg/ml	within 4 h
Evron 2008	Israel	44	50	29±7	28±5	Both	Both	NR	NR	Bolus: 20 μg lockout time: 3 min no background infusion	Loading dose: 5–10 ml of 0.2% ropivacaine CI: 10 mg/h 0.2% ropivacaine PCEA: 10 mg 0.2% ropivacaine lockout: 20 min	Within 6 h
Freeman 2015	Netherlands	687	671	31.5±5.1	31.7±4.8	Both	Both	37.8 (35.5~39.2)	37.1 (35.3~39.0)	Boluses:30 μg (20 to 40) lockout time: 3 min no background infusion	Ropivacaine or bupivacaine, or levobupivacaine plus sufentanil	during labor
Ismail 2012	Kuwait	380	380	28.4±5.5	28.6±5.5	Both	Both	39.2±1.1	39.0±1.3	Bolus: 25 μg CI:0.1–0.9 μg/kg lockout time: 1 min	Loading dose: 8 ml 0.125% levobupivacaine with 2 μg/mL fentanyl CI: 0.125% levobupivacaine with 2 μg/ml fentanyl, 8 ml/h CSEA: 2 mg levobupivacaine and 15 μg fentanyl (total 2 ml)	Within 1 h
Karadjova 2019	North Macedonia	80	75	29.9±5.2	31.3±3.8	Both	Both	NR	NR	Bolus: 0.1–1 μg/kg time: 2 min no background infusion	0.0625% bupivacaine with 2 μg/mL fentanyl	During analgesia
Li 2013	China	40	40	21~37	21~37	Both	Single	37~42	37~42	Bolus: 0.4 μg/kg, time: 2 min no background infusion	0.1% Ropivacaine + Fentanyl 2 μg/mL	during labor
Logtenberg 2017	Netherlands	203	206	31.7±3.9	31.8±4.2	Both	Both	36.1(34.3~37.6)	36.1 (33.9~37.7)	Boluses:30 μg (20 to 40) lockout time:3 min no background infusion	Loading dose: 25 mg ropivacaine 0.2% CI: 0.1% ropivacaine plus sufentanil 0.5 μg/ml	During analgesia
Stocki 2014	USA	19	19	31±5	30±6	Both	Both	NR	NR	Bolus:20–60 μg Lockout time: 2 min no background infusion	Loading dose: 0.1% bupivacaine with 50 μg fentanyl 15 ml CI: 0.1% bupivacaine with 2 μg/ml fentanyl, PCA 10 ml lockout interval: 20 min	Within 1 h
Stourac 2014	Czech Republic	12	12	27.9±3	29.4±2	Both	Both	NR	NR	Bolus:20–30 μg Lockout time:3 min no background infusion	0.125% bupivacaine with sufentanil 0.5 μg/mL	During labor
Yan 2013	China	35	35	31.6±3.8	30.9±4.1	Both	Both	NR	NR	Bolus: 0.5 μg/kg, time: 3 min no background infusion	2mg/mL ropivacaine + 2μg/mL fentanyl	During analgesia

Notes: rPCA, remifentanil patient-controlled analgesia; EA, epidural analgesia; NR, not reported

### The quality of included RCTs

**Fig 2:**
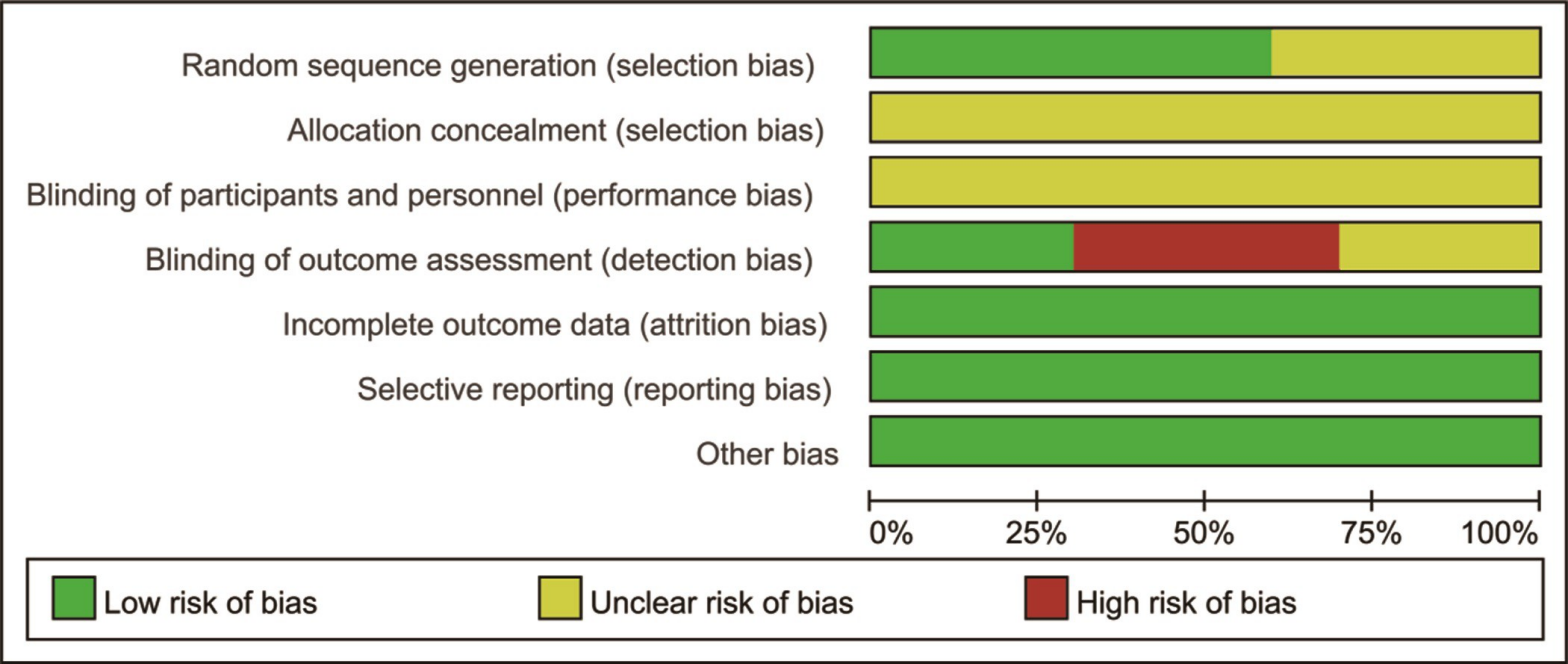
Risk of bias graph.

**Fig 3:**
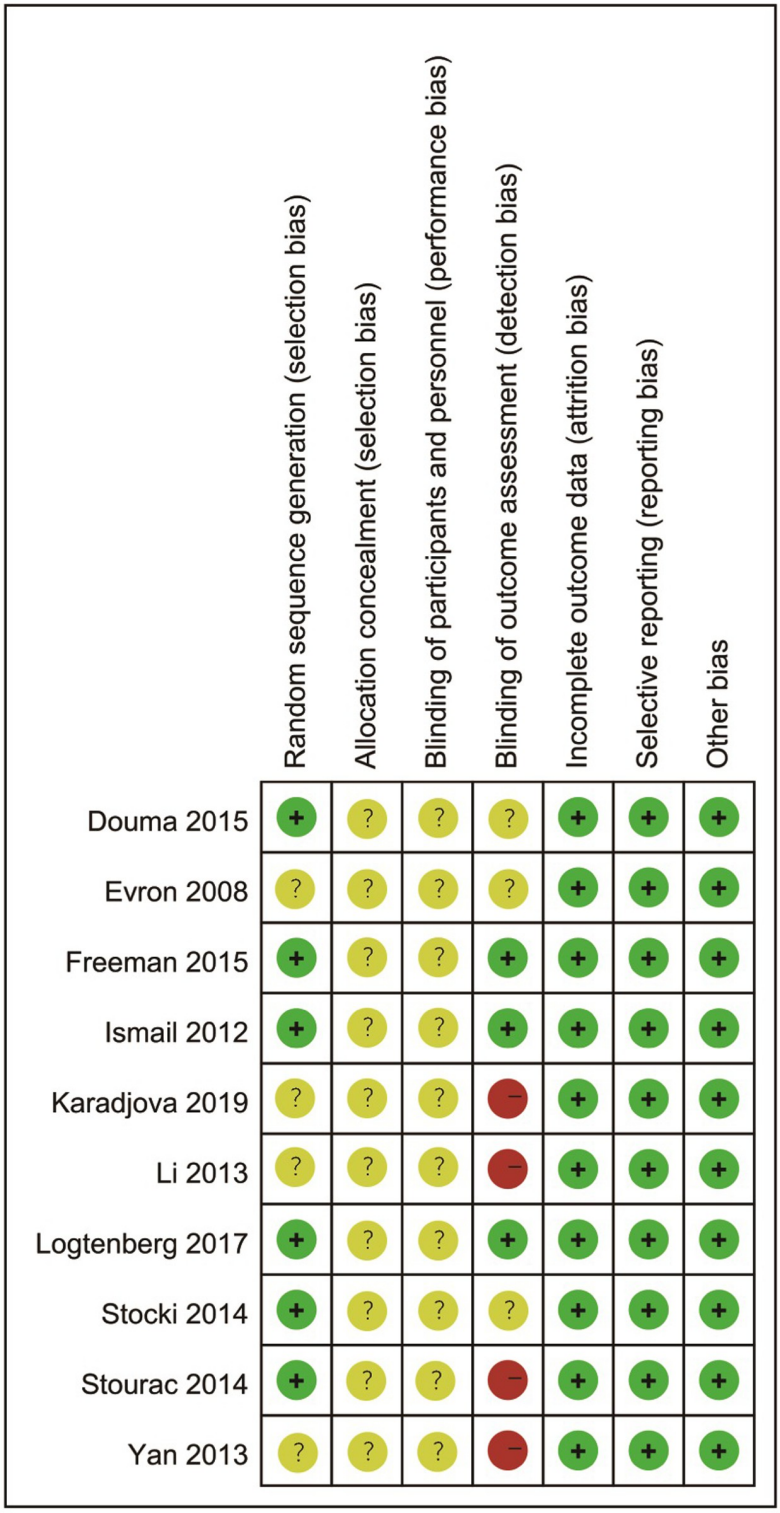
Risk of bias summary.

All domains were evaluated at low or unclear risk of bias, except the domains of blinding outcome assessment. Generally the methodological quality of included RCTs was moderate and acceptable. The quality grading of the risk of bias is presented in Figs 2 and 3.

### Meta-analysis

**Fig 4:**
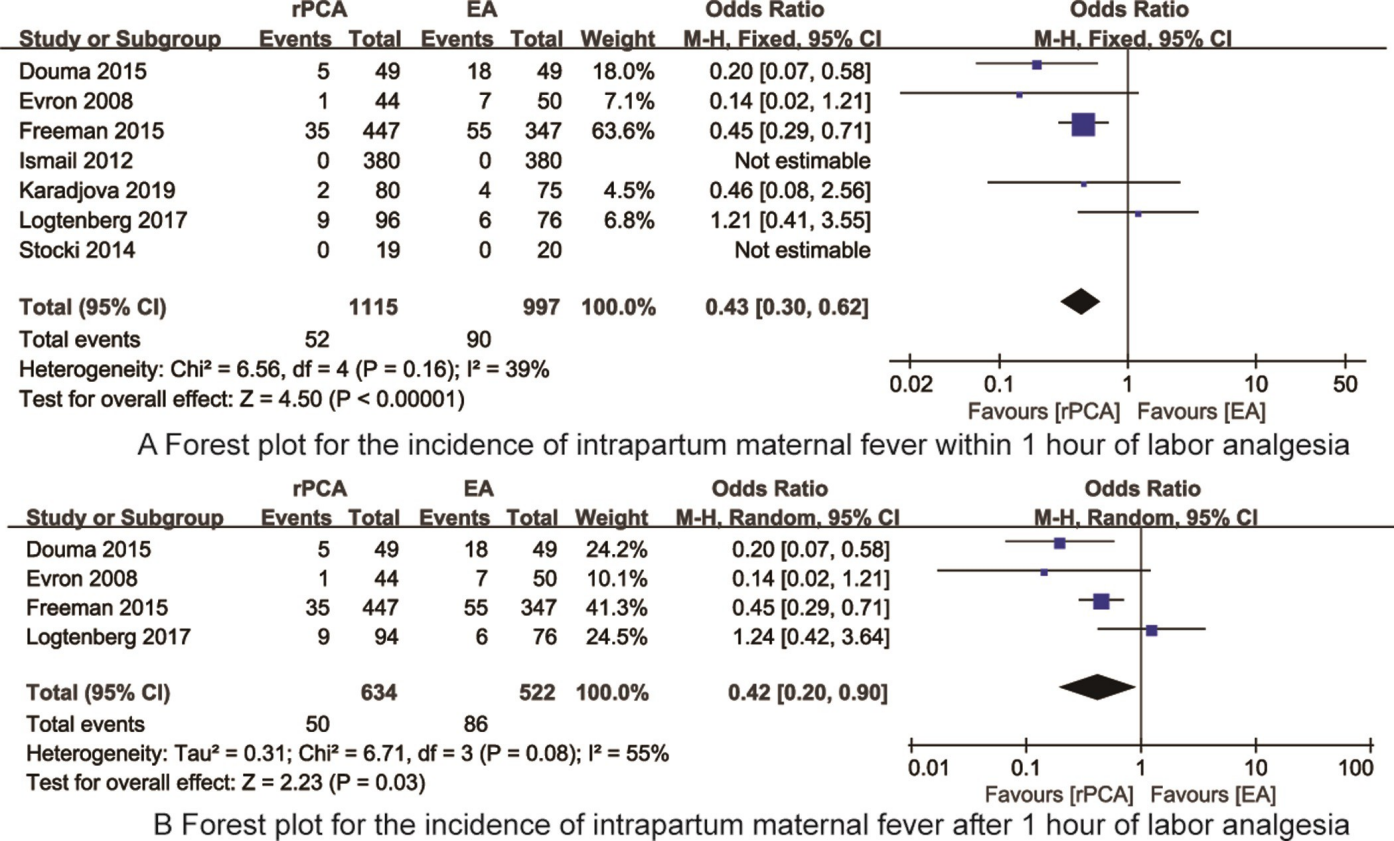
The forest plot for the incidence of intrapartum maternal fever.

*The incidence of intrapartum maternal fever within 1 hour of labor analgesia* 7 RCTs [15–17, 20–23] reported the incidence of intrapartum maternal fever within 1 hour of labor analgesia. There was no heterogeneity (I^2^ = 39%, P = 0.16) then fixed model was applied. Meta-analysis indicated that the incidence of intrapartum maternal fever within 1 hour of labor analgesia in the rPCA was significantly less than that of EA (OR = 0.43, 95%CI: 0.30~0.62, P<0.001, Fig 4A).

*The incidence of intrapartum maternal fever after 1 hour of labor analgesia* 4 RCTs [15–17, 22] reported the incidence of intrapartum maternal fever after 1 hour of labor analgesia. There was heterogeneity (I^2^ = 55%, P = 0.08) then random model was applied. Meta-analysis indicated that the incidence of intrapartum maternal fever after 1 hour of labor analgesia in the rPCA was significantly less than that of EA (OR = 0.42, 95%CI: 0.20~0.90, P = 0.03, Fig 4B).

**Fig 5:**
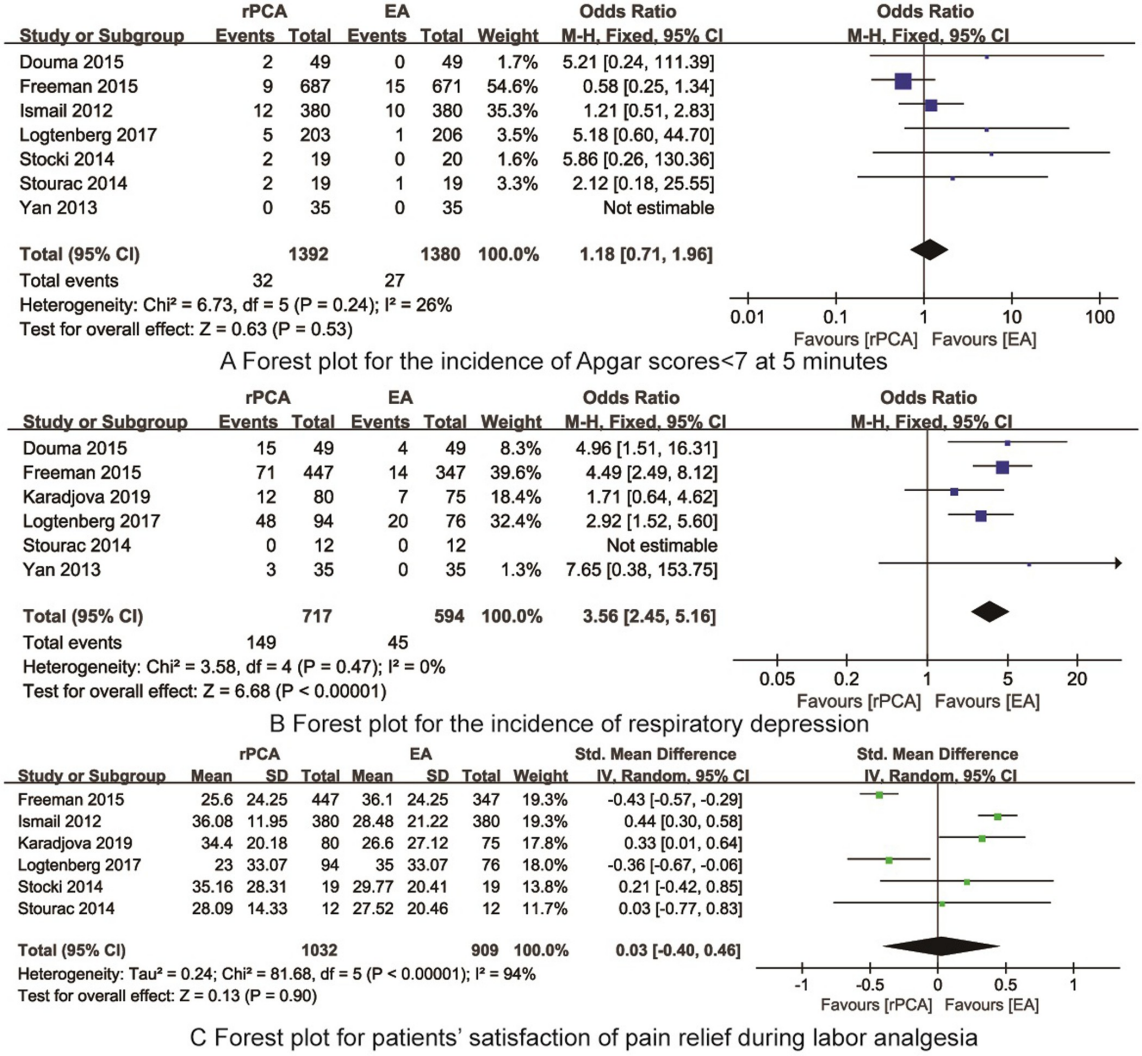
The forest plots for synthesized outcomes.

*Incidence of Apgar scores<7 at 5 minutes* 7 RCTs [15, 17, 18, 20, 22–24] reported the incidence of Apgar scores<7 at 5 minutes. There was no heterogeneity (I^2^ = 26%, P = 0.24) then fixed model was applied Meta-analysis indicated that there was no significant difference in the incidence of Apgar scores<7 at 5 minutes between rPCA and EA (OR = 1.18, 95%CI: 0.71~1.96, P = 0.53, Fig 5A).

*Incidence of respiratory depression* 6 RCTs [15, 17, 18, 21, 22, 24] reported the incidence of respiratory depression. There was no heterogeneity (I^2^ = 0%, P = 0.47) then fixed model was applied Meta-analysis indicated that the incidence of respiratory depression in the rPCA was significantly higher than that of EA (OR = 3.56, 95%CI: 2.45~5.16, P<0.001, Fig 5B).

*Patients’ satisfaction of pain relief during labor analgesia* 6 RCTs [17, 20–24] reported the patients’ satisfaction of pain relief during labor analgesia. There was heterogeneity (I^2^ = 94%, P<0.01) then random model was applied. Meta-analysis indicated that there was no significant difference in the patients’ satisfaction of pain relief during labor analgesia between rPCA and EA (SMD = 0.03, 95%CI: -0.40~0.46, P = 0.90, Fig 5C).

The synthesized outcomes of this meta-analysis are presented in S1 Table.

### Publication of bias and sensitivity analyses

The publication of bias of included RCTs was evaluated using Egger regression test. Egger regression results showed there were no significant publication biases in the synthesized outcomes (all P>0.05).

Sensitivity analyses, which evaluate the influence of one single study on the overall risk estimate by removing RCTs one by one, showed that the overall risk estimates were not substantially changed by any single RCT.

## Discussion

Remifentanil was used in obstetrics in the 1980s due to its unique pharmacological effects, and was first used for labor analgesia by PCA in 2000 [12]. Remifentanil is an ultra-short-acting μ1-receptor agonist with an onset time of 30~60s and a peak at 2.5 min [25]. It is rapidly metabolized by plasma and tissue esterases and has the advantage of a short half-life [26]. In this study, meta-analysis was used to compare the effects of rPCA and EA in labor. By increasing the sample size, the validity of the conclusions can be improved and the inconsistency of the research results can be reduced. A total of 10 RCTs were included in this meta-analysis, the results of meta-analysis show that there are no significant differences in the incidence of Apgar scores<7 at 5 minutes and the patients’ satisfaction of pain relief during labor analgesia between rPCA and EA (all P>0.05). rPCA is beneficial to reduce the incidence of intrapartum maternal fever compared with EA, but rPCA application can also increase the incidence of respiratory depression. rPCA and EA have similar pain relief effects with different characteristics, the clinical selection of rPCA and EA should be based on the actual situation of the puerpera.

Remifentanil is hydrolyzed by non-specific cholinesterase, its volume of distribution is small, the onset time is 30s, the peak effect time is 1min, the action time is 5-10min, the plasma time-dependent half-life is 3-5min, and it is rapidly cleared after drug withdrawal [27, 28]. There is no accumulation after long-term infusion, and the timing of administration is not limited [29]. The labor pain is intermittent, and the pain lags behind the uterine contractions by 10-20s [30]. In theory, the mode of PCA can make the blood concentration of remifentanil synchronize with the uterine contractions [31]. Some studies [32–34] have shown that self-controlled intravenous administration of remifentanil can effectively relieve uterine contraction pain, and compound background doses can reduce the number of compressions, improve analgesia satisfaction, have little effect on the labor process, and do not increase the rate of cesarean section.

Remifentanil is a synthetic new type of piperidine opioid μ receptor agonist, which has high-efficiency analgesia and can improve the onset speed and hydrolysis speed of anesthesia [35, 36]. The slow drug accumulation of remifentanil makes it suitable for long-term intravenous infusion. In addition, remifentanil provides rapid analgesia, can reduce excessive stress response, which can cause severe metabolic disorders, maintain normal uterine polarity, and increase maternal compliance with the delivery process, thereby shortening the labor process [31, 37, 38]. Previous studies [39, 40] have pointed out that the duration of the first, second, and third stages of labor in pregnant women in the rPCA group was lower than that in EA. Studies [41, 42] have shown that rPCA is beneficial to control stress responses such as pain and anxiety, and can inhibit excessive sympathetic nerve excitation, avoid postpartum uterine atony, and promote postpartum hemostasis. Due to the lack of data on the postpartum hemorrhage rate and volume of postpartum hemorrhage reported by the included RCTs, they cannot be included in the meta-analysis, and further research on the effects of rPCA and EA on those outcomes are needed in the future.

The clinical use of rPCA in labor must be cautioned with respiratory monitoring. Severe pain during labor can cause changes in maternal function and metabolism, reduce placental blood flow, and cause fetal hypoxia and other neonatal complications [43]. Previous studies [44, 45] have shown that rPCA has less gastrointestinal reactions, puncture site pain, and lower extremity motor block than EA. In addition, some studies [46, 47] have suggested that remifentanil can act on the sympathetic-adrenal medulla and the hypothalamic-pituitary-adrenal axis system to inhibit the release of stress hormones, thereby inhibiting the release of catechol by sympathetic nerves and regulating the estrogen levels, to improve labor compliance. Previous studies [48, 49] have shown that although remifentanil can enter the fetus through the placental barrier, its effect on neonatal respiration is low due to its low dose and rapid metabolism. Still, we should give mother and fetus complete respiratory ECG monitoring measures during rPCA application, to help quickly respond to neonatal complications and improve neonatal and maternal prognosis.

There are some limitations of this meta-analysis worth considering. Firstly, the included RCTs in his meta-analysis were all derived from published literature, and gray literatures were not searched and considered, which may have potential publication bias. Secondly, the data reported for some outcomes in the included RCTs are very limited, we could not conduct subgroup analysis to analyze the reasons for heterogeneity. Besides, the included studies did not report the important outcomes including duration of labor, which need further investigated in the future studies. Thirdly, the blinding design in the patients, intervention and evaluator is still difficult to achieve, which can lead to certain biases to the results. Future studies with larger sample size and rigorous design in different area and populations are needed to elucidate the effects and safety of rPCA.

A and EA in labor.

## Conclusions

In conclusion, rPCA can be used as an optional alternative to EA for pain relief with similar analgesic effects without reducing maternal satisfaction with pain relief and increase adverse neonatal events. However, rPCA is associated with higher risk of maternal respiratory depression during labor. Routine use of rPCA during labor must be accompanied by close respiratory monitoring. Future well-designed studies are needed to provide stronger evidence to explore the efficacy and safety of rPCA and EA in clinical labor analgesia.

## Supporting information

S1 Table The synthesized outcomes of this meta-analysis. (DOCX)

## List of abbreviations

rPCA: remifentanil patient-controlled analgesia
EA: epidural analgesia
CNKI: China National Knowledge Infrastructure
PRISMA: Preferred Reporting Items for Systematic Reviews and Meta-analysis
RCTs: randomized controlled trials
MeSH: Medical Subject Headings
SMD: standard mean difference
CI: confidence interval
OR: odd ratio
