# Supplementary material for: The efficacy and safety of remifentanil patient-controlled versus epidural analgesia in labor: A meta-analysis and systematic review
Source: PLoS One. 2022 Dec 19;17(12):e0275716. doi: 10.1371/journal.pone.0275716 (PMC9762599; doi:10.1371/journal.pone.0275716)
Supplement: S1 Table — (DOCX) [file pone.0275716.s001.docx]

Supplementary Table 1 The synthesized outcomes of this meta-analysis

| Outcome | heterogeneity (I^2^) | OR/SMD | 95%CI | P |
| --- | --- | --- | --- | --- |
| The incidence of intrapartum maternal fever within 1 hour of labor analgesia | 39% | 0.43 | 0.30~0.62, | <0.001 |
| The incidence of intrapartum maternal fever after 1 hour of labor analgesia | 55% | 0.42 | 0.20~0.90 | 0.03 |
| Incidence of Apgar scores<7 at 5 minutes | 26%, | 1.18 | 0.71~1.96 | 0.53 |
| Incidence of respiratory depression | 0% | 3.56 | 2.45~5.16 | <0.001 |
| Patients’ satisfaction of pain relief during labor analgesia | 94% | 0.03 | -0.40~0.46 | 0.90 |
